# Supplementary material for: Topology and Contribution to the Pore Channel Lining of Plasma Membrane-Embedded Shigella flexneri Type 3 Secretion Translocase IpaB
Source: mBio. 2021 Nov 23;12(6):e03021-21. doi: 10.1128/mBio.03021-21 (PMC8609354; doi:10.1128/mBio.03021-21)
Supplement: FIG S1 [file mbio.03021-21-sf001.pdf]

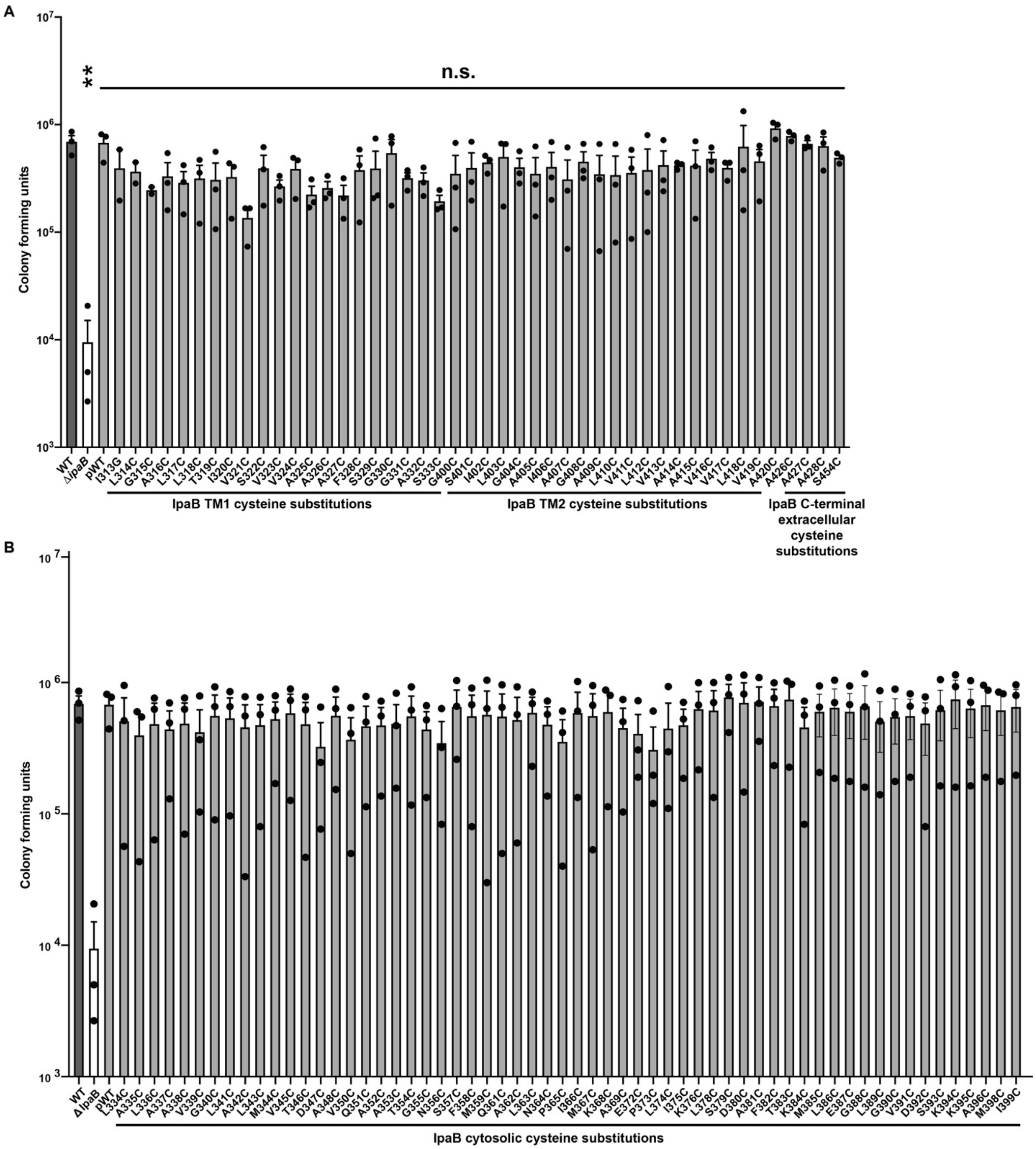
A
B
Colony forming units
10^7
10^6
10^5
10^4
10^3
n.s.
**
WT
ΔipaB
pWT
IpaB TM1 cysteine substitutions
IpaB TM2 cysteine substitutions
IpaB C-terminal extracellular cysteine substitutions
I313G L314C G315C A316C L317C L318C T319C I320C V321C S322C V323C V324C A325C A326C A327C F328C S329C G330C G331C A332C S333C
G400C S401C I402C L403C G404C A405C I406C A407C G408C A409C L410C V411C L412C V413C A414C A415C V416C V417C L418C V419C
A420C A426C A427C A428C S454C
IpaB cytosolic cysteine substitutions
L334C A335C L336C A337C A338C V339C G340C L341C A342C L343C M344C V345C T346C D347C A348C V350C Q351C A352C A353C T354C G355C N356C S357C F358C M359C Q361C A362C L363C N364C P365C I366C M367C K368C A369C E372C P373C L374C I375C K376C L378C S379C D380C A381C F382C T383C K384C M385C L386C E387C G388C L389C G390C V391C D392C S393C K394C K395C A396C M398C I399C

Supplemental Figure 1. Cysteine substitutions in IpaB do not alter *S. flexneri* invasion into host cells. Infection of HeLa monolayers with *S. flexneri* ∆*ipaB* expressing wildtype IpaB or a single IpaB cysteine substitution derivative. Positive control, wildtype *S. flexneri*. Negative control, *S. flexneri* ∆*ipaB.* Quantification of colony forming units from three independent experiments. (A) IpaB cysteine derivatives within TM1, TM2, and C-terminal extracellular domain. Means ± SEM are plotted. Black dots represent values obtained from individual experiments. **, $p<0.01$; ANOVA with Dunnett's *post hoc* test comparing colony forming units of *S. flexneri* ∆*ipaB, S. flexneri* ∆*ipaB* expressing each IpaB cysteine derivative, and *S. flexneri* ∆*ipaB* expressing WT IpaB (pWT) to colony forming units of WT *S. flexneri*. (B) IpaB cysteine derivatives within the cytosolic region. The trends observed for data in panel B do not reach statistical significance.
