# Supplementary material for: Topology and Contribution to the Pore Channel Lining of Plasma Membrane-Embedded Shigella flexneri Type 3 Secretion Translocase IpaB
Source: mBio. 2021 Nov 23;12(6):e03021-21. doi: 10.1128/mBio.03021-21 (PMC8609354; doi:10.1128/mBio.03021-21)
Supplement: FIG S2 [file mbio.03021-21-sf002.pdf]

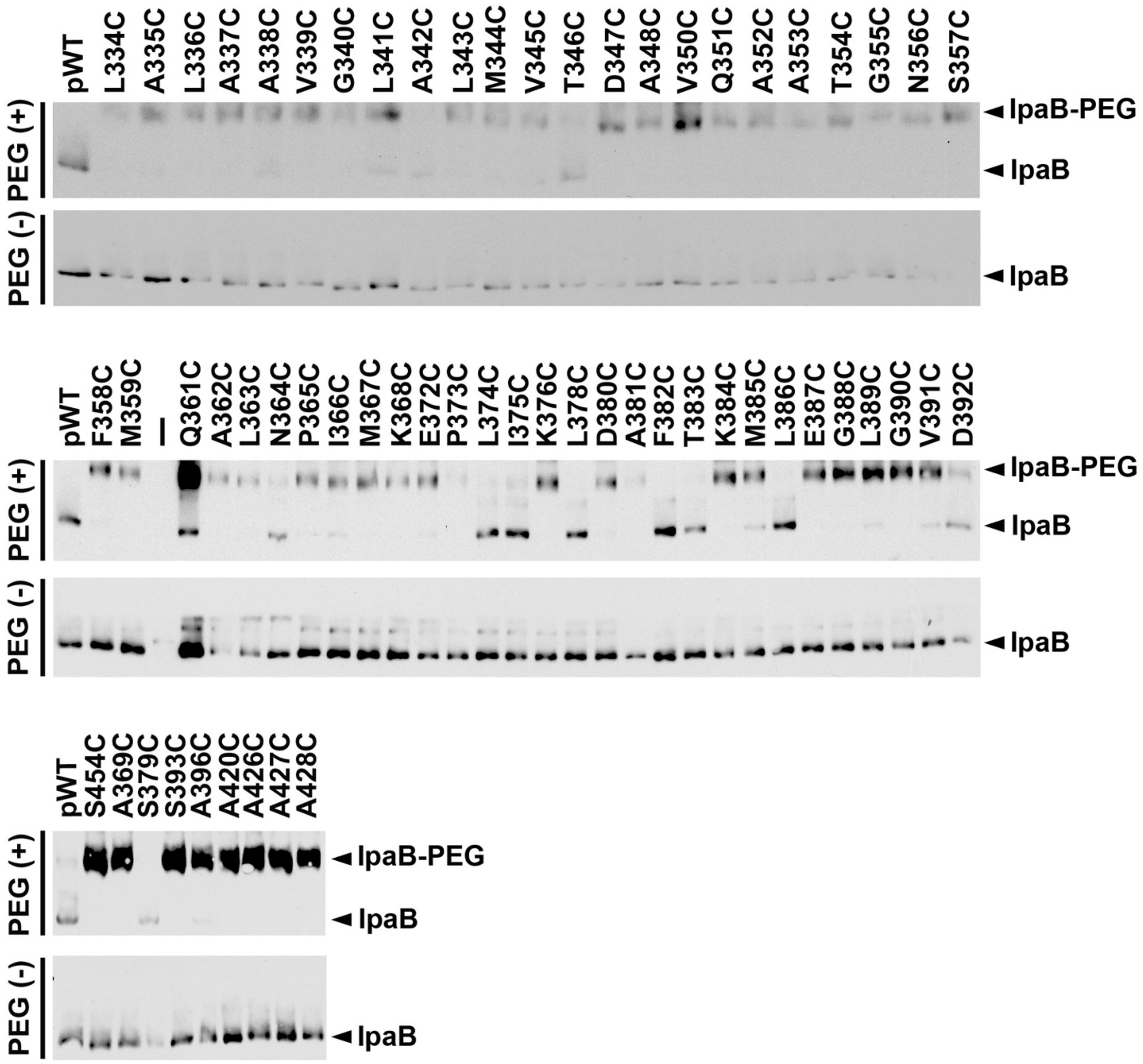

Supplemental Figure 2. In the context of soluble IpaB, most cysteine substitutions along the cytosolic domain of IpaB are accessible. Gel migration of PEG5000-maleimide labeled (IpaB-PEG) or unlabeled (IpaB) soluble IpaB in culture supernatants of indicated strains following chemical activation of type 3 secretion with Congo red. *S. flexneri ΔipaB* expressing wildtype (pWT) IpaB or a single IpaB cysteine substitution derivative. Representative western blots.
