# Supplementary material for: Topology and Contribution to the Pore Channel Lining of Plasma Membrane-Embedded Shigella flexneri Type 3 Secretion Translocase IpaB
Source: mBio. 2021 Nov 23;12(6):e03021-21. doi: 10.1128/mBio.03021-21 (PMC8609354; doi:10.1128/mBio.03021-21)
Supplement: FIG S3 [file mbio.03021-21-sf003.pdf]

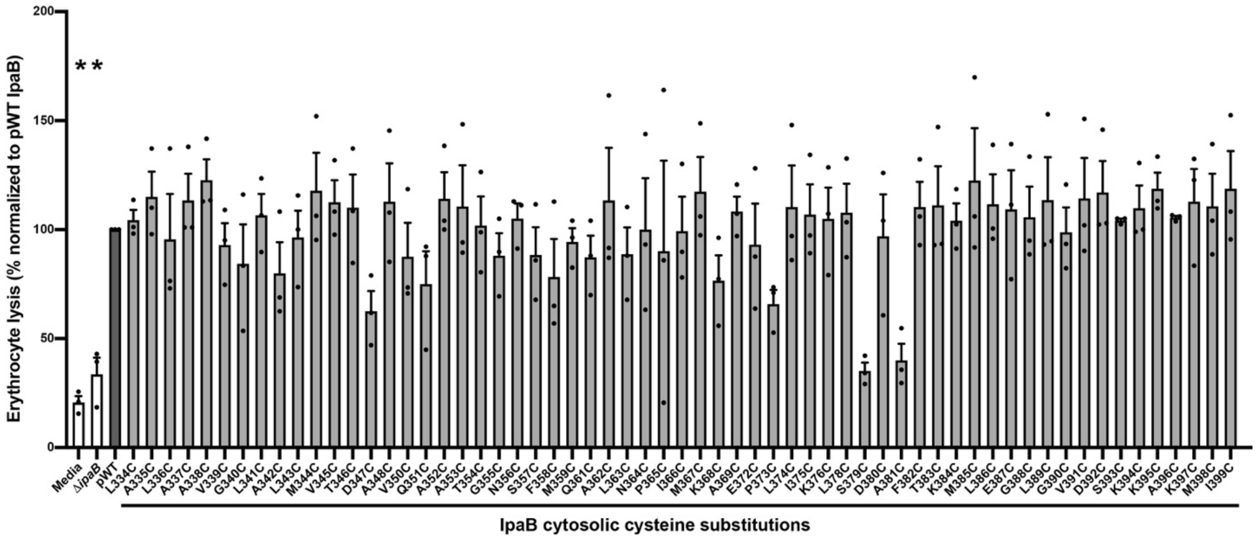

Supplemental Figure 3. Efficiency of pore formation by *S. flexneri* strains expressing single cysteine substitutions in IpaB. Quantification of hemoglobin release upon infection of sheep erythrocytes with *S. flexneri ∆ipaB*, *S. flexneri ∆ipaB* expressing wildtype IpaB (pWT) or a single cysteine substitution derivative. The abundance of hemoglobin release was quantified at A_{570} from at least three independent experiments. Means ± SEM are plotted. Black dots represent values obtained from individual experiments. *, $p<0.05$; ANOVA with Dunnett's *post hoc* test comparing each cysteine substitution mutant to *S. flexneri ∆ipaB* producing WT IpaB (pWT); the difference for each other strain is not significant.
