# Supplementary material for: Topology and Contribution to the Pore Channel Lining of Plasma Membrane-Embedded Shigella flexneri Type 3 Secretion Translocase IpaB
Source: mBio. 2021 Nov 23;12(6):e03021-21. doi: 10.1128/mBio.03021-21 (PMC8609354; doi:10.1128/mBio.03021-21)
Supplement: FIG S4 [file mbio.03021-21-sf004.pdf]

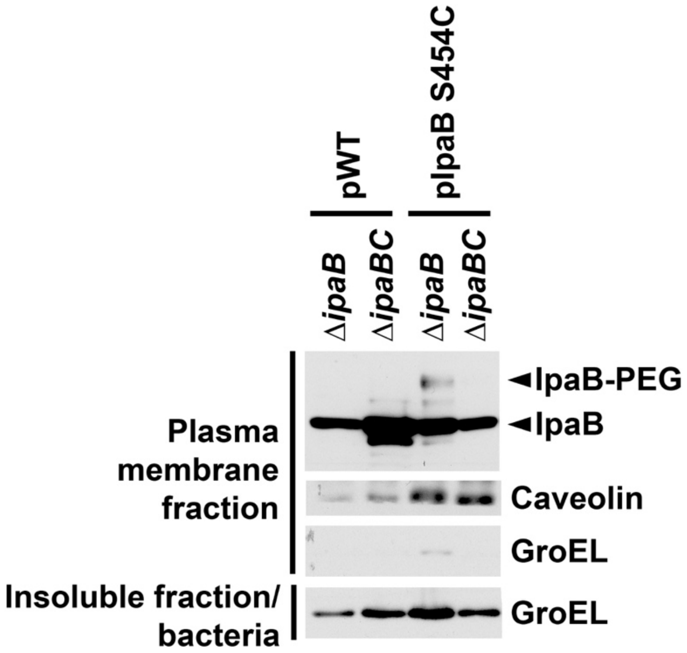

Supplemental Figure 4. In the absence of IpaC, membrane-associated IpaB C-terminal cysteine substitution does not react to PEG5000-maleimide. Gel migration of PEG5000-maleimide labeled (IpaB-PEG) or unlabeled (IpaB) IpaB in plasma membrane enriched fractions. *S. flexneri* Δ*ipaB* or Δ*ipaBC* expressing wildtype (pWT) IpaB or the IpaB cysteine substitution derivative S454C (pIpaB S454C). Positive control, *S. flexneri* Δ*ipaB* expressing IpaB S454. IpaB S454C lies in the C-terminal extracellular domain and is accessible to PEG5000-maleimide labeling in membrane inserted translocons (Fig. 3). Caveolin-1, plasma membrane marker; GroEL, bacterial cytosolic protein.
