# Supplementary material for: Topology and Contribution to the Pore Channel Lining of Plasma Membrane-Embedded Shigella flexneri Type 3 Secretion Translocase IpaB
Source: mBio. 2021 Nov 23;12(6):e03021-21. doi: 10.1128/mBio.03021-21 (PMC8609354; doi:10.1128/mBio.03021-21)
Supplement: TABLE S1 [file mbio.03021-21-st001.pdf]

## Supplemental Table

### Table S1. Strains used in this study

| Strain | Plasmid 1 | Plasmid 2 | Relevant genotype | Source or reference |
|---|---|---|---|---|
| *E. coli* DH10B | | | $F^-$ *mcr*A Δ(*mrr-hsd*RMS-*mcr*BC) φ80*lac*ZΔM15 Δ*lac*X74 *rec*A1 *end*A1 *ara*D139 Δ(*ara-leu*)7697 *gal*U *gal*K $\lambda^-$ *rps*L($Str^R$) *nup*G | Thermo Fisher (18290015) |
| *E. coli* HB101 | pIL22 | | pIL22 is pBR322 containing the pBR322 containing the AFA-I adhesin of uropathogenic E. coli K552 | (1) |
| *S. flexneri* 2457T | | | Wildtype serotype 2a | (2) |
| *S. flexneri* 2457T Δ*ipaB* | | | Deletion of *ipaB* | This study |
| *S. flexneri* 2457T Δ*acp* | | | Deletion of *acp* | This study |
| *S. flexneri* 2457T Δ*ipaB* | pDSW206-wildtype IpaB | | Δ*ipaB* expressing wildtype IpaB | This study |

| | | | | |
|---|---|---|---|---|
| *S. flexneri* 2457T Δ*ipaB* | pDSW206-IpaB C309S | | Δ*ipaB* expressing IpaB C309S | This study |
| *S. flexneri* 2457T Δ*ipaB* | pDSW206-IpaB I313C | | Δ*ipaB* expressing IpaB I313C | This study |
| *S. flexneri* 2457T Δ*ipaB* | pDSW206-IpaB L314C | | Δ*ipaB* expressing IpaB L314C | This study |
| *S. flexneri* 2457T Δ*ipaB* | pDSW206-IpaB G315C | | Δ*ipaB* expressing IpaB G315C | This study |
| *S. flexneri* 2457T Δ*ipaB* | pDSW206-IpaB A316C | | Δ*ipaB* expressing IpaB A316C | This study |
| *S. flexneri* 2457T Δ*ipaB* | pDSW206-IpaB L317C | | Δ*ipaB* expressing IpaB L317C | This study |
| *S. flexneri* 2457T Δ*ipaB* | pDSW206-IpaB L318C | | Δ*ipaB* expressing IpaB L318C | This study |
| *S. flexneri* 2457T Δ*ipaB* | pDSW206-IpaB T319C | | Δ*ipaB* expressing IpaB T319C | This study |
| *S. flexneri* 2457T Δ*ipaB* | pDSW206-IpaB I320C | | Δ*ipaB* expressing IpaB I320C | This study |
| *S. flexneri* 2457T Δ*ipaB* | pDSW206-IpaB V321C | | Δ*ipaB* expressing IpaB V321C | This study |
| *S. flexneri* 2457T Δ*ipaB* | pDSW206-IpaB S322C | | Δ*ipaB* expressing IpaB S322C | This study |
| *S. flexneri* 2457T Δ*ipaB* | pDSW206-IpaB V323C | | Δ*ipaB* expressing IpaB V323C | This study |
| *S. flexneri* 2457T Δ*ipaB* | pDSW206-IpaB V324C | | Δ*ipaB* expressing IpaB V324C | This study |
| *S. flexneri* 2457T Δ*ipaB* | pDSW206-IpaB A325C | | Δ*ipaB* expressing IpaB A325C | This study |
| *S. flexneri* 2457T Δ*ipaB* | pDSW206-IpaB A326C | | Δ*ipaB* expressing IpaB A326C | This study |
| *S. flexneri* 2457T Δ*ipaB* | pDSW206-IpaB A327C | | Δ*ipaB* expressing IpaB A327C | This study |
| *S. flexneri* 2457T Δ*ipaB* | pDSW206-IpaB F328C | | Δ*ipaB* expressing IpaB F328C | This study |

| | | | | |
|---|---|---|---|---|
| *S. flexneri* 2457T Δ*ipaB* | pDSW206-IpaB S329C | | Δ*ipaB* expressing IpaB S329C | This study |
| *S. flexneri* 2457T Δ*ipaB* | pDSW206-IpaB G330C | | Δ*ipaB* expressing IpaB G330C | This study |
| *S. flexneri* 2457T Δ*ipaB* | pDSW206-IpaB G331C | | Δ*ipaB* expressing IpaB G331C | This study |
| *S. flexneri* 2457T Δ*ipaB* | pDSW206-IpaB A332C | | Δ*ipaB* expressing IpaB A332C | This study |
| *S. flexneri* 2457T Δ*ipaB* | pDSW206-IpaB S333C | | Δ*ipaB* expressing IpaB S333C | This study |
| *S. flexneri* 2457T Δ*ipaB* | pDSW206-IpaB L334C | | Δ*ipaB* expressing IpaB L334C | This study |
| *S. flexneri* 2457T Δ*ipaB* | pDSW206-IpaB A335C | | Δ*ipaB* expressing IpaB A335C | This study |
| *S. flexneri* 2457T Δ*ipaB* | pDSW206-IpaB L336C | | Δ*ipaB* expressing IpaB L336C | This study |
| *S. flexneri* 2457T Δ*ipaB* | pDSW206-IpaB A337C | | Δ*ipaB* expressing IpaB A337C | This study |
| *S. flexneri* 2457T Δ*ipaB* | pDSW206-IpaB A338C | | Δ*ipaB* expressing IpaB A338C | This study |
| *S. flexneri* 2457T Δ*ipaB* | pDSW206-IpaB V339C | | Δ*ipaB* expressing IpaB V339C | This study |
| *S. flexneri* 2457T Δ*ipaB* | pDSW206-IpaB G340C | | Δ*ipaB* expressing IpaB G340C | This study |
| *S. flexneri* 2457T Δ*ipaB* | pDSW206-IpaB L341C | | Δ*ipaB* expressing IpaB L341C | This study |
| *S. flexneri* 2457T Δ*ipaB* | pDSW206-IpaB A342C | | Δ*ipaB* expressing IpaB A342C | This study |
| *S. flexneri* 2457T Δ*ipaB* | pDSW206-IpaB L343C | | Δ*ipaB* expressing IpaB L343C | This study |
| *S. flexneri* 2457T Δ*ipaB* | pDSW206-IpaB M344C | | Δ*ipaB* expressing IpaB M344C | This study |
| *S. flexneri* 2457T Δ*ipaB* | pDSW206-IpaB V345C | | Δ*ipaB* expressing IpaB V345C | This study |

| | | | | |
|---|---|---|---|---|
| *S. flexneri* 2457T Δ*ipaB* | pDSW206-IpaB T346C | | Δ*ipaB* expressing IpaB T346C | This study |
| *S. flexneri* 2457T Δ*ipaB* | pDSW206-IpaB D347C | | Δ*ipaB* expressing IpaB D347C | This study |
| *S. flexneri* 2457T Δ*ipaB* | pDSW206-IpaB A348C | | Δ*ipaB* expressing IpaB A348C | This study |
| *S. flexneri* 2457T Δ*ipaB* | pDSW206-IpaB V350C | | Δ*ipaB* expressing IpaB V350C | This study |
| *S. flexneri* 2457T Δ*ipaB* | pDSW206-IpaB Q351C | | Δ*ipaB* expressing IpaB Q351C | This study |
| *S. flexneri* 2457T Δ*ipaB* | pDSW206-IpaB A352C | | Δ*ipaB* expressing IpaB A352C | This study |
| *S. flexneri* 2457T Δ*ipaB* | pDSW206-IpaB A353C | | Δ*ipaB* expressing IpaB A353C | This study |
| *S. flexneri* 2457T Δ*ipaB* | pDSW206-IpaB T354C | | Δ*ipaB* expressing IpaB T354C | This study |
| *S. flexneri* 2457T Δ*ipaB* | pDSW206-IpaB G355C | | Δ*ipaB* expressing IpaB G355C | This study |
| *S. flexneri* 2457T Δ*ipaB* | pDSW206-IpaB N356C | | Δ*ipaB* expressing IpaB N356C | This study |
| *S. flexneri* 2457T Δ*ipaB* | pDSW206-IpaB S357C | | Δ*ipaB* expressing IpaB S357C | This study |
| *S. flexneri* 2457T Δ*ipaB* | pDSW206-IpaB F358C | | Δ*ipaB* expressing IpaB F358C | This study |
| *S. flexneri* 2457T Δ*ipaB* | pDSW206-IpaB M359C | | Δ*ipaB* expressing IpaB M359C | This study |
| *S. flexneri* 2457T Δ*ipaB* | pDSW206-IpaB Q361C | | Δ*ipaB* expressing IpaB Q361C | This study |
| *S. flexneri* 2457T Δ*ipaB* | pDSW206-IpaB A362C | | Δ*ipaB* expressing IpaB A362C | This study |
| *S. flexneri* 2457T Δ*ipaB* | pDSW206-IpaB L363C | | Δ*ipaB* expressing IpaB L363C | This study |
| *S. flexneri* 2457T Δ*ipaB* | pDSW206-IpaB N364C | | Δ*ipaB* expressing IpaB N364C | This study |

| | | | | |
|---|---|---|---|---|
| *S. flexneri* 2457T Δ*ipaB* | pDSW206-IpaB P365C | | Δ*ipaB* expressing IpaB P365C | This study |
| *S. flexneri* 2457T Δ*ipaB* | pDSW206-IpaB I366C | | Δ*ipaB* expressing IpaB I366C | This study |
| *S. flexneri* 2457T Δ*ipaB* | pDSW206-IpaB M367C | | Δ*ipaB* expressing IpaB M367C | This study |
| *S. flexneri* 2457T Δ*ipaB* | pDSW206-IpaB K368C | | Δ*ipaB* expressing IpaB K368C | This study |
| *S. flexneri* 2457T Δ*ipaB* | pDSW206-IpaB A369C | | Δ*ipaB* expressing IpaB A369C | This study |
| *S. flexneri* 2457T Δ*ipaB* | pDSW206-IpaB E372C | | Δ*ipaB* expressing IpaB E372C | This study |
| *S. flexneri* 2457T Δ*ipaB* | pDSW206-IpaB P373C | | Δ*ipaB* expressing IpaB P373C | This study |
| *S. flexneri* 2457T Δ*ipaB* | pDSW206-IpaB L374C | | Δ*ipaB* expressing IpaB L374C | This study |
| *S. flexneri* 2457T Δ*ipaB* | pDSW206-IpaB I375C | | Δ*ipaB* expressing IpaB I375C | This study |
| *S. flexneri* 2457T Δ*ipaB* | pDSW206-IpaB K376C | | Δ*ipaB* expressing IpaB K376C | This study |
| *S. flexneri* 2457T Δ*ipaB* | pDSW206-IpaB L378C | | Δ*ipaB* expressing IpaB L378C | This study |
| *S. flexneri* 2457T Δ*ipaB* | pDSW206-IpaB S379C | | Δ*ipaB* expressing IpaB S379C | This study |
| *S. flexneri* 2457T Δ*ipaB* | pDSW206-IpaB D380C | | Δ*ipaB* expressing IpaB D380C | This study |
| *S. flexneri* 2457T Δ*ipaB* | pDSW206-IpaB A381C | | Δ*ipaB* expressing IpaB A381C | This study |
| *S. flexneri* 2457T Δ*ipaB* | pDSW206-IpaB F382C | | Δ*ipaB* expressing IpaB F382C | This study |
| *S. flexneri* 2457T Δ*ipaB* | pDSW206-IpaB T383C | | Δ*ipaB* expressing IpaB T383C | This study |
| *S. flexneri* 2457T Δ*ipaB* | pDSW206-IpaB K384C | | Δ*ipaB* expressing IpaB K384C | This study |

| | | | | |
|---|---|---|---|---|
| *S. flexneri* 2457T Δ*ipaB* | pDSW206-IpaB M385C | | Δ*ipaB* expressing IpaB M385C | This study |
| *S. flexneri* 2457T Δ*ipaB* | pDSW206-IpaB L386C | | Δ*ipaB* expressing IpaB L386C | This study |
| *S. flexneri* 2457T Δ*ipaB* | pDSW206-IpaB E387C | | Δ*ipaB* expressing IpaB E387C | This study |
| *S. flexneri* 2457T Δ*ipaB* | pDSW206-IpaB G388C | | Δ*ipaB* expressing IpaB G388C | This study |
| *S. flexneri* 2457T Δ*ipaB* | pDSW206-IpaB L389C | | Δ*ipaB* expressing IpaB L389C | This study |
| *S. flexneri* 2457T Δ*ipaB* | pDSW206-IpaB G390C | | Δ*ipaB* expressing IpaB G390C | This study |
| *S. flexneri* 2457T Δ*ipaB* | pDSW206-IpaB V391C | | Δ*ipaB* expressing IpaB V391C | This study |
| *S. flexneri* 2457T Δ*ipaB* | pDSW206-IpaB D392C | | Δ*ipaB* expressing IpaB D392C | This study |
| *S. flexneri* 2457T Δ*ipaB* | pDSW206-IpaB S393C | | Δ*ipaB* expressing IpaB S393C | This study |
| *S. flexneri* 2457T Δ*ipaB* | pDSW206-IpaB K394C | | Δ*ipaB* expressing IpaB K394C | This study |
| *S. flexneri* 2457T Δ*ipaB* | pDSW206-IpaB K395C | | Δ*ipaB* expressing IpaB K395C | This study |
| *S. flexneri* 2457T Δ*ipaB* | pDSW206-IpaB A396C | | Δ*ipaB* expressing IpaB A396C | This study |
| *S. flexneri* 2457T Δ*ipaB* | pDSW206-IpaB M398C | | Δ*ipaB* expressing IpaB M398C | This study |
| *S. flexneri* 2457T Δ*ipaB* | pDSW206-IpaB I399C | | Δ*ipaB* expressing IpaB I399C | This study |
| *S. flexneri* 2457T Δ*ipaB* | pDSW206-IpaB G400C | | Δ*ipaB* expressing IpaB G400C | This study |
| *S. flexneri* 2457T Δ*ipaB* | pDSW206-IpaB S401C | | Δ*ipaB* expressing IpaB S401C | This study |
| *S. flexneri* 2457T Δ*ipaB* | pDSW206-IpaB I402C | | Δ*ipaB* expressing IpaB I402C | This study |

| | | | | |
|---|---|---|---|---|
| *S. flexneri* 2457T Δ*ipaB* | pDSW206-IpaB L403C | | Δ*ipaB* expressing IpaB L403C | This study |
| *S. flexneri* 2457T Δ*ipaB* | pDSW206-IpaB G404C | | Δ*ipaB* expressing IpaB G404C | This study |
| *S. flexneri* 2457T Δ*ipaB* | pDSW206-IpaB A405C | | Δ*ipaB* expressing IpaB A405C | This study |
| *S. flexneri* 2457T Δ*ipaB* | pDSW206-IpaB I406C | | Δ*ipaB* expressing IpaB I406C | This study |
| *S. flexneri* 2457T Δ*ipaB* | pDSW206-IpaB A407C | | Δ*ipaB* expressing IpaB A407C | This study |
| *S. flexneri* 2457T Δ*ipaB* | pDSW206-IpaB G408C | | Δ*ipaB* expressing IpaB G408C | This study |
| *S. flexneri* 2457T Δ*ipaB* | pDSW206-IpaB A409C | | Δ*ipaB* expressing IpaB A409C | This study |
| *S. flexneri* 2457T Δ*ipaB* | pDSW206-IpaB L410C | | Δ*ipaB* expressing IpaB L410C | This study |
| *S. flexneri* 2457T Δ*ipaB* | pDSW206-IpaB V411C | | Δ*ipaB* expressing IpaB V411C | This study |
| *S. flexneri* 2457T Δ*ipaB* | pDSW206-IpaB L412C | | Δ*ipaB* expressing IpaB L412C | This study |
| *S. flexneri* 2457T Δ*ipaB* | pDSW206-IpaB V413C | | Δ*ipaB* expressing IpaB V413C | This study |
| *S. flexneri* 2457T Δ*ipaB* | pDSW206-IpaB A414C | | Δ*ipaB* expressing IpaB A414C | This study |
| *S. flexneri* 2457T Δ*ipaB* | pDSW206-IpaB A415C | | Δ*ipaB* expressing IpaB A415C | This study |
| *S. flexneri* 2457T Δ*ipaB* | pDSW206-IpaB V416C | | Δ*ipaB* expressing IpaB V416C | This study |
| *S. flexneri* 2457T Δ*ipaB* | pDSW206-IpaB V417C | | Δ*ipaB* expressing IpaB V417C | This study |
| *S. flexneri* 2457T Δ*ipaB* | pDSW206-IpaB L418C | | Δ*ipaB* expressing IpaB L418C | This study |
| *S. flexneri* 2457T Δ*ipaB* | pDSW206-IpaB V419C | | Δ*ipaB* expressing IpaB V419C | This study |

| | | | | |
|---|---|---|---|---|
| *S. flexneri* 2457T Δ*ipaB* | pDSW206-IpaB A420C | | Δ*ipaB* expressing IpaB A420C | This study |
| *S. flexneri* 2457T Δ*ipaB* | pDSW206-IpaB A426C | | Δ*ipaB* expressing IpaB A426C | This study |
| *S. flexneri* 2457T Δ*ipaB* | pDSW206-IpaB A427C | | Δ*ipaB* expressing IpaB A427C | This study |
| *S. flexneri* 2457T Δ*ipaB* | pDSW206-IpaB A428C | | Δ*ipaB* expressing IpaB A428C | This study |
| *S. flexneri* 2457T Δ*ipaB* | pDSW206-IpaB S454C | | Δ*ipaB* expressing IpaB S454C | This study |
| *S. flexneri* 2457T Δ*ipaB* | pDSW206-wildtype IpaB | pNG162-Afa1 | Δ*ipaB* expressing wildtype IpaB and adhesin | Laboratory stock |
| *S. flexneri* 2457T Δ*ipaB* | pDSW206-IpaB I313C | pNG162-Afa1 | Δ*ipaB* expressing IpaB I313C and adhesin | This study |
| *S. flexneri* 2457T Δ*ipaB* | pDSW206-IpaB L314C | pNG162-Afa1 | Δ*ipaB* expressing IpaB L314C and adhesin | This study |
| *S. flexneri* 2457T Δ*ipaB* | pDSW206-IpaB G315C | pNG162-Afa1 | Δ*ipaB* expressing IpaB G315C and adhesin | This study |
| *S. flexneri* 2457T Δ*ipaB* | pDSW206-IpaB A316C | pNG162-Afa1 | Δ*ipaB* expressing IpaB A316C and adhesin | This study |
| *S. flexneri* 2457T Δ*ipaB* | pDSW206-IpaB L317C | pNG162-Afa1 | Δ*ipaB* expressing IpaB L317C and adhesin | This study |

| | | | | |
|---|---|---|---|---|
| *S. flexneri* 2457T Δ*ipaB* | pDSW206-IpaB L318C | pNG162-Afa1 | Δ*ipaB* expressing IpaB L318C and adhesin | This study |
| *S. flexneri* 2457T Δ*ipaB* | pDSW206-IpaB T319C | pNG162-Afa1 | Δ*ipaB* expressing IpaB T319C and adhesin | This study |
| *S. flexneri* 2457T Δ*ipaB* | pDSW206-IpaB I320C | pNG162-Afa1 | Δ*ipaB* expressing IpaB I320C and adhesin | This study |
| *S. flexneri* 2457T Δ*ipaB* | pDSW206-IpaB V321C | pNG162-Afa1 | Δ*ipaB* expressing IpaB V321C and adhesin | This study |
| *S. flexneri* 2457T Δ*ipaB* | pDSW206-IpaB S322C | pNG162-Afa1 | Δ*ipaB* expressing IpaB S322C and adhesin | This study |
| *S. flexneri* 2457T Δ*ipaB* | pDSW206-IpaB V323C | pNG162-Afa1 | Δ*ipaB* expressing IpaB V323C and adhesin | This study |
| *S. flexneri* 2457T Δ*ipaB* | pDSW206-IpaB V324C | pNG162-Afa1 | Δ*ipaB* expressing IpaB V324C and adhesin | This study |
| *S. flexneri* 2457T Δ*ipaB* | pDSW206-IpaB A325C | pNG162-Afa1 | Δ*ipaB* expressing IpaB A325C and adhesin | This study |

| | | | | |
|---|---|---|---|---|
| *S. flexneri* 2457T Δ*ipaB* | pDSW206-IpaB A326C | pNG162-Afa1 | Δ*ipaB* expressing IpaB A326C and adhesin | This study |
| *S. flexneri* 2457T Δ*ipaB* | pDSW206-IpaB A327C | pNG162-Afa1 | Δ*ipaB* expressing IpaB A327C and adhesin | This study |
| *S. flexneri* 2457T Δ*ipaB* | pDSW206-IpaB F328C | pNG162-Afa1 | Δ*ipaB* expressing IpaB F328C and adhesin | This study |
| *S. flexneri* 2457T Δ*ipaB* | pDSW206-IpaB S329C | pNG162-Afa1 | Δ*ipaB* expressing IpaB S329C and adhesin | This study |
| *S. flexneri* 2457T Δ*ipaB* | pDSW206-IpaB G330C | pNG162-Afa1 | Δ*ipaB* expressing IpaB G330C and adhesin | This study |
| *S. flexneri* 2457T Δ*ipaB* | pDSW206-IpaB G331C | pNG162-Afa1 | Δ*ipaB* expressing IpaB G331C and adhesin | This study |
| *S. flexneri* 2457T Δ*ipaB* | pDSW206-IpaB A332C | pNG162-Afa1 | Δ*ipaB* expressing IpaB A332C and adhesin | This study |
| *S. flexneri* 2457T Δ*ipaB* | pDSW206-IpaB S333C | pNG162-Afa1 | Δ*ipaB* expressing IpaB S333C and adhesin | This study |

| | | | | |
|---|---|---|---|---|
| *S. flexneri* 2457T Δ*ipaB* | pDSW206-IpaB L334C | pNG162-Afa1 | Δ*ipaB* expressing IpaB L334C and adhesin | This study |
| *S. flexneri* 2457T Δ*ipaB* | pDSW206-IpaB A335C | pNG162-Afa1 | Δ*ipaB* expressing IpaB A335C and adhesin | This study |
| *S. flexneri* 2457T Δ*ipaB* | pDSW206-IpaB L336C | pNG162-Afa1 | Δ*ipaB* expressing IpaB L336C and adhesin | This study |
| *S. flexneri* 2457T Δ*ipaB* | pDSW206-IpaB A337C | pNG162-Afa1 | Δ*ipaB* expressing IpaB A337C and adhesin | This study |
| *S. flexneri* 2457T Δ*ipaB* | pDSW206-IpaB A338C | pNG162-Afa1 | Δ*ipaB* expressing IpaB A338C and adhesin | This study |
| *S. flexneri* 2457T Δ*ipaB* | pDSW206-IpaB V339C | pNG162-Afa1 | Δ*ipaB* expressing IpaB V339C and adhesin | This study |
| *S. flexneri* 2457T Δ*ipaB* | pDSW206-IpaB G340C | pNG162-Afa1 | Δ*ipaB* expressing IpaB G340C and adhesin | This study |
| *S. flexneri* 2457T Δ*ipaB* | pDSW206-IpaB L341C | pNG162-Afa1 | Δ*ipaB* expressing IpaB L341C and adhesin | This study |

| | | | | |
|---|---|---|---|---|
| *S. flexneri* 2457T Δ*ipaB* | pDSW206-IpaB A342C | pNG162-Afa1 | Δ*ipaB* expressing IpaB A342C and adhesin | This study |
| *S. flexneri* 2457T Δ*ipaB* | pDSW206-IpaB L343C | pNG162-Afa1 | Δ*ipaB* expressing IpaB L343C and adhesin | This study |
| *S. flexneri* 2457T Δ*ipaB* | pDSW206-IpaB M344C | pNG162-Afa1 | Δ*ipaB* expressing IpaB M344C and adhesin | This study |
| *S. flexneri* 2457T Δ*ipaB* | pDSW206-IpaB V345C | pNG162-Afa1 | Δ*ipaB* expressing IpaB V345C and adhesin | This study |
| *S. flexneri* 2457T Δ*ipaB* | pDSW206-IpaB T346C | pNG162-Afa1 | Δ*ipaB* expressing IpaB T346C and adhesin | This study |
| *S. flexneri* 2457T Δ*ipaB* | pDSW206-IpaB D347C | pNG162-Afa1 | Δ*ipaB* expressing IpaB D347C and adhesin | This study |
| *S. flexneri* 2457T Δ*ipaB* | pDSW206-IpaB A348C | pNG162-Afa1 | Δ*ipaB* expressing IpaB A348C and adhesin | This study |
| *S. flexneri* 2457T Δ*ipaB* | pDSW206-IpaB V350C | pNG162-Afa1 | Δ*ipaB* expressing IpaB V350C and adhesin | This study |

| | | | | |
|---|---|---|---|---|
| *S. flexneri* 2457T Δ*ipaB* | pDSW206-IpaB Q351C | pNG162-Afa1 | Δ*ipaB* expressing IpaB Q351C and adhesin | This study |
| *S. flexneri* 2457T Δ*ipaB* | pDSW206-IpaB A352C | pNG162-Afa1 | Δ*ipaB* expressing IpaB A352C and adhesin | This study |
| *S. flexneri* 2457T Δ*ipaB* | pDSW206-IpaB A353C | pNG162-Afa1 | Δ*ipaB* expressing IpaB A353C and adhesin | This study |
| *S. flexneri* 2457T Δ*ipaB* | pDSW206-IpaB T354C | pNG162-Afa1 | Δ*ipaB* expressing IpaB T354C and adhesin | This study |
| *S. flexneri* 2457T Δ*ipaB* | pDSW206-IpaB G355C | pNG162-Afa1 | Δ*ipaB* expressing IpaB G355C and adhesin | This study |
| *S. flexneri* 2457T Δ*ipaB* | pDSW206-IpaB N356C | pNG162-Afa1 | Δ*ipaB* expressing IpaB N356C and adhesin | This study |
| *S. flexneri* 2457T Δ*ipaB* | pDSW206-IpaB S357C | pNG162-Afa1 | Δ*ipaB* expressing IpaB S357C and adhesin | This study |
| *S. flexneri* 2457T Δ*ipaB* | pDSW206-IpaB F358C | pNG162-Afa1 | Δ*ipaB* expressing IpaB F358C and adhesin | This study |

| | | | | |
|---|---|---|---|---|
| *S. flexneri* 2457T Δ*ipaB* | pDSW206-IpaB M359C | pNG162-Afa1 | Δ*ipaB* expressing IpaB M359C and adhesin | This study |
| *S. flexneri* 2457T Δ*ipaB* | pDSW206-IpaB Q361C | pNG162-Afa1 | Δ*ipaB* expressing IpaB Q361C and adhesin | This study |
| *S. flexneri* 2457T Δ*ipaB* | pDSW206-IpaB A362C | pNG162-Afa1 | Δ*ipaB* expressing IpaB A362C and adhesin | This study |
| *S. flexneri* 2457T Δ*ipaB* | pDSW206-IpaB L363C | pNG162-Afa1 | Δ*ipaB* expressing IpaB L363C and adhesin | This study |
| *S. flexneri* 2457T Δ*ipaB* | pDSW206-IpaB N364C | pNG162-Afa1 | Δ*ipaB* expressing IpaB N364C and adhesin | This study |
| *S. flexneri* 2457T Δ*ipaB* | pDSW206-IpaB P365C | pNG162-Afa1 | Δ*ipaB* expressing IpaB P365C and adhesin | This study |
| *S. flexneri* 2457T Δ*ipaB* | pDSW206-IpaB I366C | pNG162-Afa1 | Δ*ipaB* expressing IpaB I366C and adhesin | This study |
| *S. flexneri* 2457T Δ*ipaB* | pDSW206-IpaB M367C | pNG162-Afa1 | Δ*ipaB* expressing IpaB M367C and adhesin | This study |

| | | | | |
|---|---|---|---|---|
| *S. flexneri* 2457T Δ*ipaB* | pDSW206-IpaB K368C | pNG162-Afa1 | Δ*ipaB* expressing IpaB K368C and adhesin | This study |
| *S. flexneri* 2457T Δ*ipaB* | pDSW206-IpaB A369C | pNG162-Afa1 | Δ*ipaB* expressing IpaB A369C and adhesin | This study |
| *S. flexneri* 2457T Δ*ipaB* | pDSW206-IpaB E372C | pNG162-Afa1 | Δ*ipaB* expressing IpaB E372C and adhesin | This study |
| *S. flexneri* 2457T Δ*ipaB* | pDSW206-IpaB P373C | pNG162-Afa1 | Δ*ipaB* expressing IpaB P373C and adhesin | This study |
| *S. flexneri* 2457T Δ*ipaB* | pDSW206-IpaB L374C | pNG162-Afa1 | Δ*ipaB* expressing IpaB L374C and adhesin | This study |
| *S. flexneri* 2457T Δ*ipaB* | pDSW206-IpaB I375C | pNG162-Afa1 | Δ*ipaB* expressing IpaB I375C and adhesin | This study |
| *S. flexneri* 2457T Δ*ipaB* | pDSW206-IpaB K376C | pNG162-Afa1 | Δ*ipaB* expressing IpaB K376C and adhesin | This study |
| *S. flexneri* 2457T Δ*ipaB* | pDSW206-IpaB L378C | pNG162-Afa1 | Δ*ipaB* expressing IpaB L378C and adhesin | This study |

| | | | | |
|---|---|---|---|---|
| *S. flexneri* 2457T Δ*ipaB* | pDSW206-IpaB S379C | pNG162-Afa1 | Δ*ipaB* expressing IpaB S379C and adhesin | This study |
| *S. flexneri* 2457T Δ*ipaB* | pDSW206-IpaB D380C | pNG162-Afa1 | Δ*ipaB* expressing IpaB D380C and adhesin | This study |
| *S. flexneri* 2457T Δ*ipaB* | pDSW206-IpaB A381C | pNG162-Afa1 | Δ*ipaB* expressing IpaB A381C and adhesin | This study |
| *S. flexneri* 2457T Δ*ipaB* | pDSW206-IpaB F382C | pNG162-Afa1 | Δ*ipaB* expressing IpaB F382C and adhesin | This study |
| *S. flexneri* 2457T Δ*ipaB* | pDSW206-IpaB T383C | pNG162-Afa1 | Δ*ipaB* expressing IpaB T383C and adhesin | This study |
| *S. flexneri* 2457T Δ*ipaB* | pDSW206-IpaB K384C | pNG162-Afa1 | Δ*ipaB* expressing IpaB K384C and adhesin | This study |
| *S. flexneri* 2457T Δ*ipaB* | pDSW206-IpaB M385C | pNG162-Afa1 | Δ*ipaB* expressing IpaB M385C and adhesin | This study |
| *S. flexneri* 2457T Δ*ipaB* | pDSW206-IpaB L386C | pNG162-Afa1 | Δ*ipaB* expressing IpaB L386C and adhesin | This study |

| | | | | |
|---|---|---|---|---|
| *S. flexneri* 2457T Δ*ipaB* | pDSW206-IpaB E387C | pNG162-Afa1 | Δ*ipaB* expressing IpaB E387C and adhesin | This study |
| *S. flexneri* 2457T Δ*ipaB* | pDSW206-IpaB G388C | pNG162-Afa1 | Δ*ipaB* expressing IpaB G388C and adhesin | This study |
| *S. flexneri* 2457T Δ*ipaB* | pDSW206-IpaB L389C | pNG162-Afa1 | Δ*ipaB* expressing IpaB L389C and adhesin | This study |
| *S. flexneri* 2457T Δ*ipaB* | pDSW206-IpaB G390C | pNG162-Afa1 | Δ*ipaB* expressing IpaB G390C and adhesin | This study |
| *S. flexneri* 2457T Δ*ipaB* | pDSW206-IpaB V391C | pNG162-Afa1 | Δ*ipaB* expressing IpaB V391C and adhesin | This study |
| *S. flexneri* 2457T Δ*ipaB* | pDSW206-IpaB D392C | pNG162-Afa1 | Δ*ipaB* expressing IpaB D392C and adhesin | This study |
| *S. flexneri* 2457T Δ*ipaB* | pDSW206-IpaB S393C | pNG162-Afa1 | Δ*ipaB* expressing IpaB S393C and adhesin | This study |
| *S. flexneri* 2457T Δ*ipaB* | pDSW206-IpaB K394C | pNG162-Afa1 | Δ*ipaB* expressing IpaB K394C and adhesin | This study |

| | | | | |
|---|---|---|---|---|
| *S. flexneri* 2457T Δ*ipaB* | pDSW206-IpaB K395C | pNG162-Afa1 | Δ*ipaB* expressing IpaB K395C and adhesin | This study |
| *S. flexneri* 2457T Δ*ipaB* | pDSW206-IpaB A396C | pNG162-Afa1 | Δ*ipaB* expressing IpaB A396C and adhesin | This study |
| *S. flexneri* 2457T Δ*ipaB* | pDSW206-IpaB M398C | pNG162-Afa1 | Δ*ipaB* expressing IpaB M398C and adhesin | This study |
| *S. flexneri* 2457T Δ*ipaB* | pDSW206-IpaB I399C | pNG162-Afa1 | Δ*ipaB* expressing IpaB I399C and adhesin | This study |
| *S. flexneri* 2457T Δ*ipaB* | pDSW206-IpaB G400C | pNG162-Afa1 | Δ*ipaB* expressing IpaB G400C and adhesin | This study |
| *S. flexneri* 2457T Δ*ipaB* | pDSW206-IpaB S401C | pNG162-Afa1 | Δ*ipaB* expressing IpaB S401C and adhesin | This study |
| *S. flexneri* 2457T Δ*ipaB* | pDSW206-IpaB I402C | pNG162-Afa1 | Δ*ipaB* expressing IpaB I402C and adhesin | This study |
| *S. flexneri* 2457T Δ*ipaB* | pDSW206-IpaB L403C | pNG162-Afa1 | Δ*ipaB* expressing IpaB L403C and adhesin | This study |

| | | | | |
|---|---|---|---|---|
| *S. flexneri* 2457T Δ*ipaB* | pDSW206-IpaB G404C | pNG162-Afa1 | Δ*ipaB* expressing IpaB G404C and adhesin | This study |
| *S. flexneri* 2457T Δ*ipaB* | pDSW206-IpaB A405C | pNG162-Afa1 | Δ*ipaB* expressing IpaB A405C and adhesin | This study |
| *S. flexneri* 2457T Δ*ipaB* | pDSW206-IpaB I406C | pNG162-Afa1 | Δ*ipaB* expressing IpaB I406C and adhesin | This study |
| *S. flexneri* 2457T Δ*ipaB* | pDSW206-IpaB A407C | pNG162-Afa1 | Δ*ipaB* expressing IpaB A407C and adhesin | This study |
| *S. flexneri* 2457T Δ*ipaB* | pDSW206-IpaB G408C | pNG162-Afa1 | Δ*ipaB* expressing IpaB G408C and adhesin | This study |
| *S. flexneri* 2457T Δ*ipaB* | pDSW206-IpaB A409C | pNG162-Afa1 | Δ*ipaB* expressing IpaB A409C and adhesin | This study |
| *S. flexneri* 2457T Δ*ipaB* | pDSW206-IpaB L410C | pNG162-Afa1 | Δ*ipaB* expressing IpaB L410C and adhesin | This study |
| *S. flexneri* 2457T Δ*ipaB* | pDSW206-IpaB V411C | pNG162-Afa1 | Δ*ipaB* expressing IpaB V411C and adhesin | This study |

| | | | | |
|---|---|---|---|---|
| *S. flexneri* 2457T Δ*ipaB* | pDSW206-IpaB L412C | pNG162-Afa1 | Δ*ipaB* expressing IpaB L412C and adhesin | This study |
| *S. flexneri* 2457T Δ*ipaB* | pDSW206-IpaB V413C | pNG162-Afa1 | Δ*ipaB* expressing IpaB V413C and adhesin | This study |
| *S. flexneri* 2457T Δ*ipaB* | pDSW206-IpaB A414C | pNG162-Afa1 | Δ*ipaB* expressing IpaB A414C and adhesin | This study |
| *S. flexneri* 2457T Δ*ipaB* | pDSW206-IpaB A415C | pNG162-Afa1 | Δ*ipaB* expressing IpaB A415C and adhesin | This study |
| *S. flexneri* 2457T Δ*ipaB* | pDSW206-IpaB V416C | pNG162-Afa1 | Δ*ipaB* expressing IpaB V416C and adhesin | This study |
| *S. flexneri* 2457T Δ*ipaB* | pDSW206-IpaB V417C | pNG162-Afa1 | Δ*ipaB* expressing IpaB V417C and adhesin | This study |
| *S. flexneri* 2457T Δ*ipaB* | pDSW206-IpaB L418C | pNG162-Afa1 | Δ*ipaB* expressing IpaB L418C and adhesin | This study |
| *S. flexneri* 2457T Δ*ipaB* | pDSW206-IpaB V419C | pNG162-Afa1 | Δ*ipaB* expressing IpaB V419C and adhesin | This study |

| | | | | |
|---|---|---|---|---|
| *S. flexneri* 2457T Δ*ipaB* | pDSW206-IpaB A420C | pNG162-Afa1 | Δ*ipaB* expressing IpaB A420C and adhesin | This study |
| *S. flexneri* 2457T Δ*ipaB* | pDSW206-IpaB A426C | pNG162-Afa1 | Δ*ipaB* expressing IpaB A426C and adhesin | This study |
| *S. flexneri* 2457T Δ*ipaB* | pDSW206-IpaB A427C | pNG162-Afa1 | Δ*ipaB* expressing IpaB A427C and adhesin | This study |
| *S. flexneri* 2457T Δ*ipaB* | pDSW206-IpaB A428C | pNG162-Afa1 | Δ*ipaB* expressing IpaB A428C and adhesin | This study |
| *S. flexneri* 2457T Δ*ipaB* | pDSW206-IpaB S454C | pNG162-Afa1 | Δ*ipaB* expressing IpaB S454C and adhesin | This study |
| *S. flexneri* 2457T Δ*ipaBC* | pDSW206-wildtype IpaB | | Δ*ipaBC* expressing wildtype IpaB | This study |
| *S. flexneri* 2457T Δ*ipaBC* | pDSW206-IpaB S454C | | Δ*ipaBC* expressing IpaB S454C | This study |

## References


1. Labigne-Roussel AF, Lark D, Schoolnik G, Falkow S. Cloning and expression of an afimbrial adhesin (AFA-I) responsible for P blood group-independent, mannose-resistant hemagglutination from a pyelonephritic Escherichia coli strain. Infect Immun. 1984;46(1):251-9. Epub 1984/10/01. doi: 10.1128/iai.46.1.251-259.1984. PubMed PMID: 6148308; PMCID: PMC261465.

2. Labrec EH, Schneider H, Magnani TJ, Formal SB. Epithelial Cell Penetration as an Essential Step in the Pathogenesis of Bacillary Dysentery. J Bacteriol. 1964;88(5):1503-18. Epub 1964/11/01. doi: 10.1128/jb.88.5.1503-1518.1964. PubMed PMID: 16562000; PMCID: PMC277436.
